# Thrombosis Risk Associated with Head and Neck Cancer: A Review

**DOI:** 10.3390/ijms20112838

**Published:** 2019-06-11

**Authors:** Pierre Haen, Diane Mege, Lydie Crescence, Françoise Dignat-George, Christophe Dubois, Laurence Panicot-Dubois

**Affiliations:** 1 Aix Marseille Univ, INSERM 1263, INRA, Center for CardioVascular and Nutrition Research (C2VN), 27 Boulevard Jean Moulin, 13385 Marseille, France; pierre.haen@yahoo.fr (P.H.); DIANE.MEGE@ap-hm.fr (D.M.); Lydie.crescence@univ-amu.fr (L.C.); Francoise.dignat-george@univ-amu.fr (F.D.-G.); christophe.dubois@univ-amu.fr (C.D.); 2 Department of Maxillo-Facial Surgery, Army Training Hospital, Laveran, 13013 Marseille, France; 3 Department of Digestive Surgery, Timone University Hospital, AP-HM, 13005 Marseille, France; 4 Laboratoire d’Hématologie, Centre Hospitalo-Universitaire Conception, 385 Boulevard Baille, 13385 Marseille, France

**Keywords:** Head and neck neoplasms, venous thromboembolism, squamous cell carcinoma, thrombosis, cancer

## Abstract

Venous thromboembolism (VTE) is a common complication for cancer patients. VTE-associated risk varies according to the type of tumor disease. Head and neck cancer is a common cancer worldwide, and most tumors are squamous cell carcinomas due to tobacco and alcohol abuse. The risk of VTE associated with head and neck (H&N) cancer is considered empirically low, but despite the high incidence of H&N cancer, few data are available on this cancer; thus, it is difficult to state the risk of VTE. Our review aims to clarify this situation and tries to assess the real VTE risk associated with H&N cancer. We report that most clinical studies have concluded that there is a very low thrombosis risk associated with H&N cancer. Even with the biases that often exist, this clinical review seems to confirm that the risk of VTE was empirically hypothesized. Furthermore, we highlight that H&N cancer has all the biological features of a cancer associated with a high thrombosis risk, including a strong expression of procoagulant proteins, modified thrombosis/fibrinolysis mechanisms, and secretions of procoagulant microparticles and procoagulant cytokines. Thus, this is a paradoxical situation, and some undiscovered mechanisms that could explain this clinical biological ambivalence might exist.

## 1. Introduction

The association between cancer and venous thromboembolism (VTE) has been known since its historical description by Trousseau [1] and Bouillaud [2] in the 19th century. Since then, several studies have established that thrombosis is a common complication for cancer patients, and it has been estimated that patients with cancer have an approximately sevenfold higher risk of VTE than those without cancer [3]. Several biological mechanisms have been highlighted and focus on a hypercoagulable state induced by malignant cells [4], including: expression of procoagulant protein [5], release of procoagulant microparticles [6], induced secretion of procoagulant inflammatory cytokines [7], and support of a prothrombotic state on platelets, endothelial cells or leucocytes [8]. The VTE risk varies in accordance with cancer type, location, stage and histological grade and classification [9]. Additionally, factors related to cancer management, such as surgery, chemotherapy, radiotherapy, hormonal therapy, hospitalization with long-term bed rest, and indwelling venous catheters, further increase the VTE risk [9,10]. VTE disease is the second cause of mortality in cancer patients [10], following the malignant disease itself. Treatment and prophylaxis of VTE are crucial parts of the global management of patients with cancer. However, even if prophylaxis has a positive effect on the emergence of VTE [11,12], it has not been clearly demonstrated that VTE prophylaxis has an impact on the cancer patients’ mortality [11,13]. The main incriminated factor is that antithrombotic treatments are not risk-free and can be responsible for life-threatening hemorrhage, especially in at-risk patients with tumors [12]. VTE prophylaxis can be challenging; therefore, it is essential to perform a thorough assessment of the VTE risk based initially on the cancer characteristics.

Although its incidence has declined in the last ten years, head and heck (H&N) cancer is still among the most common cancers worldwide. With approximately 500,000 new cases and 150,000 deaths per year in the world, H&N cancer ranks between the 8th and 10th most frequently occurring cancer, depending on the country [14,15,16]. H&N cancer includes oral cavity cancer (lip, tongue, mucosa and gingivae), pharyngeal cancer (oropharynx, nasopharynx, hypopharynx), laryngeal cancer, thyroid cancer and some cancer of the upper part of the esophagus. Oral cavity cancer is the most frequent localization [17]. More than 90% of H&N cancers are squamous cell carcinomas (SCCs) [18]. Historically, head and neck squamous cell carcinoma (H&N SCC) risk factors are tobacco use and alcohol abuse, but human papillomavirus has recently been identified as a risk factor, especially for tongue localization [19]. Despite H&N SCC being a common disease, it is difficult to determine the risk of thrombosis. Indeed, the available data in the literature seem to be contradictory, with most studies suggesting a poor or nonexistent thrombosis risk associated with H&N SCC and some studies supporting a notable associated risk. Moreover, few specific data are available, and confounding factors are often present in those studies, which contributes to why the conclusions remain unclear.

Our review aims to clarify the available findings on this topic, especially through biological studies that can support clinical observations.

## 2. The lack of Clinical Evidence

Thrombosis risk associated with H&N cancer is empirically rated to be very weak or, in fact, be nonexistent [20,21]. However, an overview of the literature tells us that the situation is not black and white, and it seems difficult to obtain evidence-based proof, notably because of numerous biases. We looked at the major clinical studies that reported VTE associated with H&N cancer and assessed the risk of thrombosis. A literature review has been carried out with three main focuses:

Analysis of studies that assessed the incidence of cancer diagnosis following a VTE and those that evaluated the risk according to cancer localization. We focused on H&N cancer incidence and found that the incidence of H&N cancer corresponded between 0% and 1.45% of all diagnosed cancers. H&N cancer was almost ranked as the least common cancer following VTE. The data are listed in Table 1.

According to an analysis of studies that assessed the incidence of thromboembolism in patients diagnosed with cancer according to its localization, we studied the specific risk of VTE following H&N cancer and compared the incidence of VTE with H&N cancer to that of other cancer localizations. We found an estimated incidence of VTE in H&N cancer patients from 0.16% to (more than) 3.125%. The thrombosis risk associated with H&N cancer was one of the lowest of the studied cancers, except in the Paneesha et al. study, where H&N cancer was ranked second behind pancreas cancer [22]. The data are listed in Table 2.

In an analysis of studies that were specifically interested in thrombosis associated with H&N cancer, especially following surgery, we found an incidence of VTE in H&N cancer patients ranging from 0% to 26.3%. The data are listed in Table 3. 

No matter how the data are reported, many of those studies concluded that there was a very low thrombosis risk associated with head and neck carcinoma, with almost always an evaluated risk defined as the lowest of all studied cancer localizations. However, it is notable that some studies found different results and reported a high thrombosis risk associated with H&N cancer. These consisted largely of studies specifically oriented on the incidence of VTE and H&N cancer, as well as the study from Paneesha et al., which was mentioned above. In contrast, Levitan and colleagues [33] and Stein et al. [36] reported rates of VTE incidence in H&N cancer patients (0.16% and 0.6%, respectively) that were lower than in their corresponding control group of hospitalized patients without oncological diseases (0.57% and 1%, respectively). This may suggest that H&N cancer might have a protective effect on VTE [49]. All these facts revealed that there is a somewhat considerable lack of clarity on the incidence of VTE, which seems difficult to efficiently determine.

A possible explanation is that the studies on this topic are subject to confounding factors and that “the perfect” study would probably be impossible to conduct.

The first bias is that most of the studies were interested in H&N cancer; even if the majority of tumors had the same characteristics, the tumors are heterogeneous, which should lead to some differences in the thrombosis risk. In fact, H&N cancer is caused by large squamous cell carcinoma (SCC), principally from the oral cavity, oropharynx, hypopharynx and larynx. All of these cancers are relatively close, especially in terms of histopathological features and clinical evolution [50,51]. In contrast, some cancers, such as thyroid tumors or salivary gland tumors, are also included in the H&N cancer group and are clearly different in terms of the histopathological features, clinical evolution and thrombosis-associated risk. Indeed, it has been clearly shown that adenocarcinomas (thyroid or salivary gland) have a higher thrombosis-associated risk than squamous cell carcinomas [52,53]. The same applies to nasopharyngeal carcinoma, which is a specific tumor that is very undifferentiated and is far from the typical H&N SCC that has a well-known high thrombosis-associated risk [54].

Second, some risk factors of H&N cancer are also independent risk factors for an elevated risk of venous thromboembolism. Tobacco use and an age older than 50 years have clearly been identified to be risk factors for H&N SCC, but these factors are also independent risk factors for VTE [18,55]. The same is true for the male sex, which is also associated with both diseases [56].

Lastly, most reported studies included patients who underwent treatment for their malignancies, and many of these treatments are also known to be independent risk factors of venous thromboembolism. These factors include surgical treatment, a favored treatment for H&N cancer that is often involves long procedures, especially in cases of simultaneous reconstruction [47,57,58]. These factors also include chemotherapy, which has been clearly identified as an independent risk factor for venous thromboembolism [59,60].

In short, this clinical study review has shown that the thrombosis risk associated with H&N cancer seems to be low, as it has been empirically believed. However, some studies had contradictory outcomes, and we showed that confounding factors exist. Thus, there exists no strong clinical proof to make conclusions about the true risk associated with H&N cancer or H&N SCC. The continuation of this work will consist of finding biological arguments that could help to stratify this risk.

## 3. Clinical Practice and Recommendations

Because of a lack of clinical evidence, there is no specific evidence that can serve as a basis for recommendations about the clinical practice of VTE management in H&N cancer patients. However, general strong evidence-based recommendations are available about VTE management in patients with cancer, especially those reported by the American Society of Clinical Oncology (ASCO), which can be considered as references [11,61]. Some good clinical practices for managing VTE in surgical H&N patients are also available and are given by societies of H&N surgeons or ENTs [62,63].

### 3.1. VTE Diagnosis

The diagnostic strategies for VTE associated with cancer do not differ from the strategies used to diagnose VTE of other causes. Some of the diagnostic tests include lower limb vein compression ultrasonography for deep venous thrombosis and computed tomography angiography to test for pulmonary embolism; although the ventilation perfusion lung scan is no longer used, this method can be an alternative test [64]. It is not recommended to systematically search for VTE in cancer patients but to only screen patients with clinical signs and risk factors [65]. Some clinical scoring systems could be used to help accomplish this task. The blood D-dimer test would also be useful, particularly because a negative result would permit the exclusion of a VTE diagnosis [64,66].

Ultrasonography of the neck, which can be completed by computed tomography angiography or magnetic resonance imaging with angiography, is indicated in cases of neck thrombosis, especially in cases of internal jugular vein thrombosis [67].

### 3.2. VTE Treatment

Low-molecular-weight heparin (LMWH) is the preferred initial anticoagulation treatment for diagnosed VTE associated with cancer. LMWH is also indicated for long-term anticoagulant treatment. In all cases, treatment that lasts for longer than six months must be discussed and reserved for patients with a high risk of VTE recurrence. The recommended treatment following the first VTE diagnosis should continue for three to six months [68]. The benefit/risk balance must be evaluated before treatment, particularly in regard to the bleeding risk [61]. Vitamin K antagonists (VKAs) can be an alternative for long-term treatment if LMWH is not available or is contraindicated or if the malignancy stabilized and the treatment was completed [11]. Incidentally discovered VTE must be treated similarly [69]. The same treatment would also be indicated for neck vein thrombosis [67].

### 3.3. VTE Prophylaxis

According to the clinical guidelines given by the ASCO, most hospitalized patients with active cancer require thromboprophylaxis throughout hospitalization. This recommendation does not apply to ambulatory patients or patients undergoing minor procedures, unless the patient has a high risk of VTE. The VTE risk is determined using validated assessment tools, including assessments of the kind and localization of cancer, treatment strategy and some individual and biological characteristics (age, comorbidities, platelet counts, etc.) [11,61,70]. Some assessment tools have also been validated for H&N cancer surgery, such as the Caprini risk assessment model [58,62,71]. In cases of major surgery for cancer, which is almost always the case for H&N cancer, the ASCO clearly recommends thromboprophylaxis; the thromboprophylaxis must be started before surgery and be continued for 7–10 days after surgery. The use of LMWH is recommended, and the hemorrhagic risk must be evaluated before starting treatment. H&N surgeons and ENT societies recommend mechanical prophylaxis for VTE for all patients with VTE risk. The same societies also recommend pharmacological thromboprophylaxis by LMWH. Pharmacological thromboprophylaxis is not systematically provided by certain teams without the occurrence of additional VTE events following H&N cancer surgery [44,58,62,72,73]. This issue is addressed in the previous paragraph.

## 4. Biological/Fundamental Research Contributions

### 4.1. Background

The existence of a biological interrelation between cancer and the hemostatic system is widely recognized for most cancers, especially for colon, pancreas, ovarian and lung cancer. Different biological mechanisms have been shown to explain thrombosis associated with cancer: expression of hemostatic proteins [74] or adhesion platelet molecules by tumor cells [75], production of procoagulant microparticles (MP) by tumor and/or host cells [76], inflammation [77], and the presence of other molecules such as some proangiogenic factors (VEGF, βTGF) [78].

These mechanisms have been less studied for H&N cancer, most likely because the associated clinical thrombosis risk is considered low; however, existing data seem to show that a similar interrelation might also exist for this kind of cancer. As stated above, thrombosis risk is dependent on the histological features of the tumor, which is why we limited our review to H&N SCC, the more representative kind of tumor in H&N cancer.

### 4.2. H&N SCC and the Proteins Related to Aggregation and Coagulation Mechanisms

#### 4.2.1. Tissue Factor/Tissue Factor Pathway Inhibitor (and Thrombin)

Tissue factor (TF), also called factor III or thromboplastin, is a transmembrane glycoprotein that plays a central role in blood clotting. Physiologically, TF triggers blood coagulation by binding factor VII and converting factor VII to its active form, VIIa. The TF/VIIa complex activates factor X and ultimately facilitates thrombin and fibrin production. TF is normally present in connective tissue but not in circulating blood. TF can be expressed by endothelial cells, monocytes and neutrophils after induction [79]. Tissue factor pathway inhibitor (TFPI) is a plasma serine protease inhibitor, which is a natural powerful inhibitor of the factor X and TF/VIIa complex. TFPI is normally synthetized by vascular endothelial cells. TFPI could also be expressed by fibroblasts, smooth muscle cells and macrophages [80]. The thrombotic profile of a cell or a tissue is in fact related to the TFPI/TF balance. In addition, TF and TFPI are also involved in maintaining pathological conditions, especially in cancer [81]. It has been showed that TF is involved in cancer angiogenesis, invasion, growth, metastasis and cancer-associated thrombosis by triggering blood clotting. The ectopic expression of TF has been detected in several cancers, including gastric cancer [82], pancreatic cancer [83] and breast cancer [84]. The inappropriate expression of TF in tumor cells is due to the activation of the pro-oncogene EGFR, which enhances TF expression [85]. This relationship seems to also apply for H&N SCC; the immunofluorescence study by Wojtsukiewicz et al. showed strong expression of TF on cells from human laryngeal SCC [86]. Christensen et al. found the same conclusion with cells from human oral squamous cell carcinoma (OSCC) using the same imaging method [87]. Chang et al. also found elevated TF expression in SAS cells, a human cell line of OSCC, by increasing mRNA coding for TF. Moreover, their study showed that SAS cells could induce platelet aggregation in vitro (also called TCIPA, or tumor-cell induced platelet aggregation) by a mechanism that directly involves TF [88]. Using flow cytometry, Welsh et al. demonstrated that several H&N SCC cell lines expressed TF at a more elevated level than other cell lines reputed to have high-level TF expression. The authors have also shown that the level of TF expression was directly correlated with the procoagulant cell capacity in vitro [89]. Another procoagulant mechanism involving TF was highlighted by Adesanya et al. They showed that the in vitro production of specific microvesicles from H&N SCC cell lines could facilitate TF expression by endothelial cells by inducing blood clotting and platelet aggregation [90]. This mechanism is certainly implicated in the strong expression of TF in H&N SCC pericarcinomatous tissue, as shown by Wang et al. in their immunohistological study conducted on human laryngeal SCC [91].

Even if it had been studied less, TFPI would have a role in cancer. The expression of TFPI has also been observed in some tumors and cancer cell lines, such as those from breast, pancreatic and colorectal cancer [81,92]. In contrast to TF, TFPI may have a beneficial effect on cancer cells and decreases the invasive potential of the disease, especially by inhibiting serine proteases such as matrix metalloproteases and plasmins involved in determining the aggressiveness of cancer [93]. Wojtukiewicz et al. showed that cells from human laryngeal SCC did not express high levels TFPI [86]; moreover, they showed in another study that the TFPI expression level in laryngeal SCC tissue was decreased compared to that in normal laryngeal tissue [94]. TFPI was recently recognized as a tumor-suppressor gene in melanoma [95], and it has even been used as a gene therapy target to successfully treat laryngeal squamous cell carcinoma in mice [96].

Taken together, these facts suggest that the TF/TFPI balance is clearly in benefit of TF and that H&N SCC should be associated with a high risk of VTE. Practically, we did not find this result in our clinical review about H&N SCC and its associated VTE thrombosis risk, which was rather considered very low. The high expression of TF seems to be insufficient to increase the thrombosis risk associated with H&N cancer, as has also been shown for other cancer localization [97]. High TF expression does, however, explain why prothrombin and fibrin are present in tumor and peritumor sites of HNSCC cancer; prothrombin and fibrin are indicators of local activation of blood coagulation [98]. This might also explain the frequency of venous thrombosis in the areas adjacent to H&N SCC (internal jugular vein thrombosis); the thrombosis may occur by platelet activation/aggregation due to direct contact with tumor cells or by endothelial cells activated by tumor cell microvesicles [99,100].

#### 4.2.2. The Fibrinolysis and Plasmin-Plasminogen System

The blood fibrinolytic system relies on an inactive proenzyme, plasminogen, that can be converted to an active form, plasmin. Plasmin is a serine protease that degrades the final product of the coagulation system, fibrin, which is directly implicated in VTE. Under normal conditions, the balance between fibrinolysis and the coagulation system results in an increase or decrease of each, which has consequences on the risk of VTE. Thus, the diminution of the fibrinolytic system activities would increase the VTE risk [101]. Plasminogen is synthetized by hepatocytes and is widely present in the blood circulation under normal conditions. Plasminogen activation consists of two other serine proteases, tissue plasminogen activator (tPA) and urokinase plasminogen activator (uPA), along with its cellular receptor (urokinase plasminogen activator receptor—uPAR). tPA is synthetized by endothelial cells and is activated by fibrin. uPA is synthetized by many cell types, especially kidney cells, and is activated by factor XII. The activity of uPA is enhanced when uPA is fixed to its receptor uPAR, which is expressed by many different cells. Plasminogen activator inhibitor (PAI) is the most important inhibitor of the fibrinolytic system and blocks the activation of tPA and uPA. The serine protease of PAI exists in two forms: one constitutive form, PAI-1, and one form only present during pregnancy and some hematological diseases, PAI-2. The two other inhibitors that exist are α-2-antiplasmin and thrombin-activated fibrinolysis inhibitor (TAFI). These inhibitors directly inhibit fibrin degradation by plasmin.

Kosugi et al. were the first to show modifications of the fibrinolysis system in patients with H&N SCC based on blood examinations. They also suggested the down-regulation of the fibrinolysis system which might cause an elevated VTE risk [102]. Later, Jagielska et al. conducted a study based on the blood examination of 32 patients with advanced H&N SCC and reached the same conclusion; moreover, they showed a significant elevation of levels of PAI-1 in the blood, which might explain the down regulation of the fibrinolysis system and the potential increase in the VTE risk [103]. Hundsdorfer and colleagues, using enzyme-linked immunosorbent assays (ELISA), analyzed OSCC in 79 patients to compare uPA and PAI-1 levels between tumor tissue and normal tissue. They found a global elevation of both proteases, without a true superiority of either the activator or inhibitor of plasminogen [104]. Serpa et al. also found elevated uPA levels in a similar study conducted on human tongue SCC and showed that SCC cells had an elevation in the expression of uPAR [105]. Baker et al. conducted a more complete study and showed an elevation in uPA, uPAR, PAI-1 and PAI-2 expression in human OSCC tumor tissue. They also showed a correlation between protease levels and the aggressiveness of the tumor [106]. This relationship is not surprising because the role of plasmin (matrix degradation, metalloprotease activation) has been clearly shown in facilitating the progression and metastasis processes of H&N SCC [107,108]. We did not find any studies about TAFI expression and H&N cancer; however, it has been shown that some polymorphisms of the TAFI gene can have a protective effect for OSCC [109].

In summary, despite the trend of fibrinolysis downregulation that seemingly exists in H&N cancer, which would enhance the incidence of associated thrombosis, it is difficult to definitively define this phenomenon. In fact, there could be a global elevation of plasminogen activator (tPA, uPA) and inhibitor (PAI) levels, which can balance with each other without consequence on thrombosis risk.

#### 4.2.3. Others Coagulation/Aggregation Factors

##### Thromboxane A2 and Prostacyclin

Thromboxane A2 (TXA2) and prostacyclin (PGI2) are two products formed from arachidonic acid by cyclooxygenase. TXA2 is an activator of platelet aggregation, and its action is due to the enhanced concentration of Ca2+ in platelets following TXA2 binding on its own receptor. PGI2 has the opposite effect, with a strong inhibition of platelet aggregation through the stimulation of adenylate cyclase that then leads to an increase in cyclic AMP and a diminution of Ca2+ concentration in the platelets. An elevation of TXA2 or diminution of PGI2 levels would enhance thrombosis risk, and such a mechanism could be involved in thrombosis associated with colon cancer [110,111].

Using radioimmunoassays of human laryngeal SCC, Pinto et al. showed in their study that there was a significant elevation of TXA2 production in tumor and peritumor tissues compared with in normal tissue [112]. Using the same technical method, Slotman found an elevated plasma TXA2 concentration in patients with H&N SCC. The author also found that none of these patients had elevated plasma PGI2 concentrations [113]. Camacho et al. found the same results in their study using mRNA PGI2 pathway enzyme analysis conducted on human H&N SCC; moreover, they showed an association between a decrease in PGI2 production and poor prognosis of the disease [114]. In brief, the metabolism of TXA2 and PGI2 might be affected in H&N cancer patients, with a balance in favor of TXA2, which could be responsible for an increase in thrombosis risk.

##### Podoplanin

Podoplanin is a mucin-type transmembrane glycoprotein that is specifically expressed in lymphatic endothelial cells but not in normal blood endothelial cells. Podoplanin has the ability to induce direct platelet activation via the platelet-receptor CLEC-2, and the role of podoplanin in cancer-associated thrombosis has been mainly reported for brain tumors [115]. However, the overexpression of podoplanin and its potential role in cancer-associated thrombosis has also been shown in other kinds of tumors, such as lung [116] or breast [117]. H&N SCC is also be associated with a high expression of podoplanin. Yuan et al. showed an overexpression of podoplanin in human oral and hypopharyngeal SCC tissue in their immunohistological studies [118]. Martin-Villar et al. found the same conclusions in their genetic and immunohistological study of OSCC using human genetic material transfected into mice. Moreover, they highlighted a potential role of podoplanin expression in OSCC and platelet activation [119]. All of these studies also showed a strong correlation between cancer prognosis and podoplanin, especially its existence in cervical lymph nodes [120]. Thus, aggressive H&N cancer with high podoplanin expression can be associated with an elevated associated thrombosis risk.

##### Von Willebrand Factor

Von Willebrand factor (vWF) is a plasma glycoprotein synthesized in endothelial cells and megakaryocytes. vWF promotes the adhesion of platelets to subendothelial connective tissue and to endothelial cells in cases of blood vessel damage. vWF is also a carrier protein for factor VIII. Some studies have demonstrated increased vWF plasma levels in cancer cells with a potential implication in thrombosis-associated risk [121,122]. Sweeney and colleagues first highlighted the elevation of vWF plasma levels in patients with extended H&N SCC [123]. Paczuski et al. conducted a similar study in patients with laryngeal SCC and concluded that patients with extended cancer or with metastatic lymph nodes had a high plasmatic level of vWF [124]. As in the Sweeney study, the authors did not find any significant difference in vWF plasma levels between patients with small or moderate cancer and patients without disease. Similar to podoplanin levels, vWF plasmatic levels are enhanced with the aggressiveness and size of H&N tumors, and then associated with a potential elevation of the thrombosis risk.

##### Thrombomodulin

Thrombomodulin (TM) is a glycoprotein that was originally identified on the vascular endothelium and characterized as a major natural endothelial anticoagulant. TM plays a role as a cofactor for thrombin binding and mediates protein C activation and inhibits thrombin activity. TM is physiologically expressed on various cells, such as megakaryocytes, platelets, monocytes, macrophages, and different squamous and mesothelial cells [125]. It has been shown that TM is also expressed on various cancers such as lung or pancreas cancer [126,127] and that elevated TM expression is associated with a decrease in the aggressiveness of the tumor. The same findings have been shown for H&N cancer. Tabata et al. showed the expression of TM in human OSCC tissue in their study based on immunohistological examinations. Moreover, they showed a relationship between TM level, the risk of lymph node metastasis and the survival rate. A high level of TM might be associated with a better prognosis [128]. Gaspar et al. found the same conclusions in their study about human laryngeal and pharyngeal SCC [129]. These findings suggest that aggressive tumors with low TM levels are associated with a high risk of associated thrombosis.

Our research has also explored coagulation inhibitors (antithrombin, heparin cofactor II, S protein, etc.) as well as other coagulation/aggregation related proteins, especially platelet adhesion molecules (platelet integrin α2β3, p-selectin, cadherin, etc.), but has not found any convincing results. Taking all these separate pieces of information together, we believe that biological findings would be quite in favor of an elevated risk of thrombosis associated with H&N cancer.

### 4.3. H&N SCC and Microparticles

Microparticles (MPs) are small membrane vesicles ranging from 0.1 to 1 µm in size and are formed by budding from the plasma membrane. MPs are released following cell activation, injury or apoptosis. Circulating MPs play important roles in physiological and pathological conditions, especially in cancer-associated thrombosis [130]. MPs can be released directly from cancer cells but also by other cell types (platelets, endothelial cells, neutrophils, etc.) after being induced by tumor cells. The procoagulant activity of MPs could not only be due to TF membrane expression but also to the presence of phosphatidylserine, which provides a negatively charged surface to support the assembly of coagulation complexes. Even though we did not find any studies that specifically highlighted the plasmatic presence of TF-bearing MP from H&N SCC cells, this presence is suggested by some studies; this occurrence is easy to imagine since we showed that H&N SCC cells had high TF expression levels and H&N SCC patients have facial areas with rich vascularization.

Ren et al. showed elevated levels of circulating platelet-derived MPs in 63 patients with OSCC compared to in healthy volunteers and compared to in patients with infected benign cysts of the jaw. The authors also showed an increased level of total circulating MPs in patients with OSCC. Moreover, they highlighted that circulating MPs from OSCC patient plasma had procoagulant activity and that the circulating MPs reduced coagulation time of normal plasma vitro when they were added. However, no modifications of activated thromboplastin time (APTT) and prothrombin time (PT) was found in OSCC patients [131].

As discussed above, Adesanya et al. have shown that H&N SCC cells could produce in vitro microvesicles with TF-dependent procoagulant activity. The authors also showed that H&N SCC-derived microvesicles could activate umbilical vein endothelial cells with TF surface expression and a procoagulant profile [90]. Sierko et al. has shown a high level of plasma TF-bearing endothelial MPs in their study conducted in 16 patients with H&N SCC treated by radiotherapy. However, they did not find any abnormalities in the hemostatic system in those patients [132].

These findings about MPs support the arguments for an elevated thrombosis risk associated with H&N SCC, but an effective MP-dependent thrombosis risk is supported by the balance between pro and anticoagulant MPs [133]. However, we did not find any reports about anticoagulant MPs in H&N cancer.

### 4.4. H&N SCC, Inflammation and Angiogenesis

Cancer is subject to an inflammatory response that is responsible for a procoagulant state. On one hand, this is due to the direct modulation of platelet function (activation) and, on the other hand, to procoagulant cytokine secretion [134]. The most well-defined pro-inflammatory cytokines that have been shown to exert pro-thrombotic effects are tumor necrosis factor alpha (TNFα) and interleukin-1 (IL-1). TNFα and IL-1 can induce the expression of TF and von Willebrand factor (vWF) on vascular endothelial cells. It has also been shown that interleukin-6 (IL-6) has a pro-thrombotic effect by expressing TF, vWF and factor VIII but also by downregulating the expression of antithrombotic molecules such as antithrombin III, protein S and thrombomodulin [4,135]. Similar to other cancers, H&N SCC is subject to systemic inflammation responses and secretions of cytokines by immune cells. Gallo et al. showed a significantly higher potential for TNFα secretion by monocytes from patients with OSCC than from control patients without tumors [136]. Parks et al. evaluated that the proper production of TNFα by tumor cells in vitro using cells issued from tumors of 9 patients with H&N SCC [137]. In vivo TNFα secretion by H&N SCC cells was confirmed by Nakano et al. in their study based on immunohistological analysis and more recently by Scheff et al. in their study conducted in mice [138,139]. The same findings are also applicable for IL-1 and IL-6, and Gallo et al. and Tsai et al. showed an increase in vitro in interleukin secretion by monocytes from patients with H&N SCC [140,141]. Thomas et al. showed that murine OSCC cell lines were able to directly express IL-1 and IL-6 in vitro [142]. Wang et al. confirmed these findings in vivo in humans and showed that this secretion contributed to a high level of IL-1 and IL-6 in the circulating blood and in the tumor peri-environment [143].

Other cytokines, especially those implicated in angiogenesis associated with tumor development, are known to have a prothrombotic effect. Vascular endothelial growth factor (VEGF) is a potent angiogenic cytokine implicated in tumor vasculogenesis. It has been shown that VEGF has a procoagulant effect by inducing TF expression by macrophages [78,144]. Chen et al. showed that OSCC cell lines freshly cultured from human tumors are able to secrete VEGF in vitro [145]. Johnston et al. and Eisma et al. demonstrated that high VEGF expression existed in OSCC tumors and in OSCC tumor environments in vivo in humans [146,147]. Granulocyte-macrophage colony-stimulating factor (GM-CSF) is a cellular growing factor shown to have potential procoagulant activity due to its stimulation of vWF production [148]. The expression of GM-CSF by tumor cells and its presence in the tumor environment of H&N SCC has also been shown [142,149]. Taken together, these findings suggest that there is elevated production/secretion of procoagulant cytokines in H&N cancer, which is again supports the argument for an effective associated thrombosis risk.

## 5. H&N SCC, Thrombosis and Cancer Progression

As we have shown, it seems that a local prothrombotic area associated with H&N cancer exists, which can lead to platelet activation/aggregation and thrombus generation in the tumor environment. Platelets are a large reservoir of biomolecules that are stored in granules. Upon activation, especially in tumor cells, platelets can release their granule contents. Through this action, it has been shown that platelets could play several roles in the progression of malignancies [150]. Platelet alpha granules are full of growth and angiogenic factors, such as vascular epithelial growth factor (VEGF), epidermal growth factor (EGF), platelet-derived growth factor (PDGF) and transforming growth factor (TGFβ). VEGF, as mentioned above, is a growth factor involved in the induction of vasculogenesis and angiogenesis, which are both needed for tumor growth [151]. The angiogenic role of VEGF has been demonstrated in various types of cancers, and the potential role of VEGF in the progression of OSCC has also been reported [152]. The potential effect of VEGF on bone invasion by OSCC cells has also been shown [153]. EGF is a powerful mitogen growth factor that has an important effect on OSCC cells that overexpress the EGF receptor. Platelet EGF promotes invasion, migration, and epithelial mesenchymal transition in OSCC cells [154]. These roles are why anti-EGF receptor therapy can work as an OSCC treatment [155]. PDGF are peptides that signal through cell-surface receptor tyrosine kinases (PDGFRs) and activate various cellular functions, such as proliferation, growth and differentiation. The effect of PDGF on different kinds of cancers has been shown in breast and some digestive cancers. The effect of PDGF on OSCC cells has also been reported, especially for tongue SCC [156,157]. The role of TGFβ, another growth factor, is far less clear. Indeed, according to the stage of cancer, TGF can promote or inhibit tumor progression. This effect has been shown for squamous cell carcinoma, including H&N SCC [158]. In short, with the possible exception of TGFβ, platelet secretion products can have a positive effect on tumor progression and invasion. Moreover, it should be noted that the platelet and fibrinogen coat around the tumor might protect the tumor against the cytotoxic activity of natural killer cells, as Palumbo et al. showed in their study about breast cancer in mice [159]. In addition, platelet aggregation around cancer cells can also be useful in protecting cancer cells against high shear stress in the bloodstream [160].

Nonetheless, even if there are no reports yet, one might believe that the very low rate of VTE associated with H&N cancer would reflect the scarcity of distant metastases in this kind of tumor; in contrast, certain tumors that have a high thrombosis risk also have a high risk of metastasis, such as pancreatic cancer [161].

## 6. Conclusions

**Figure 1:**
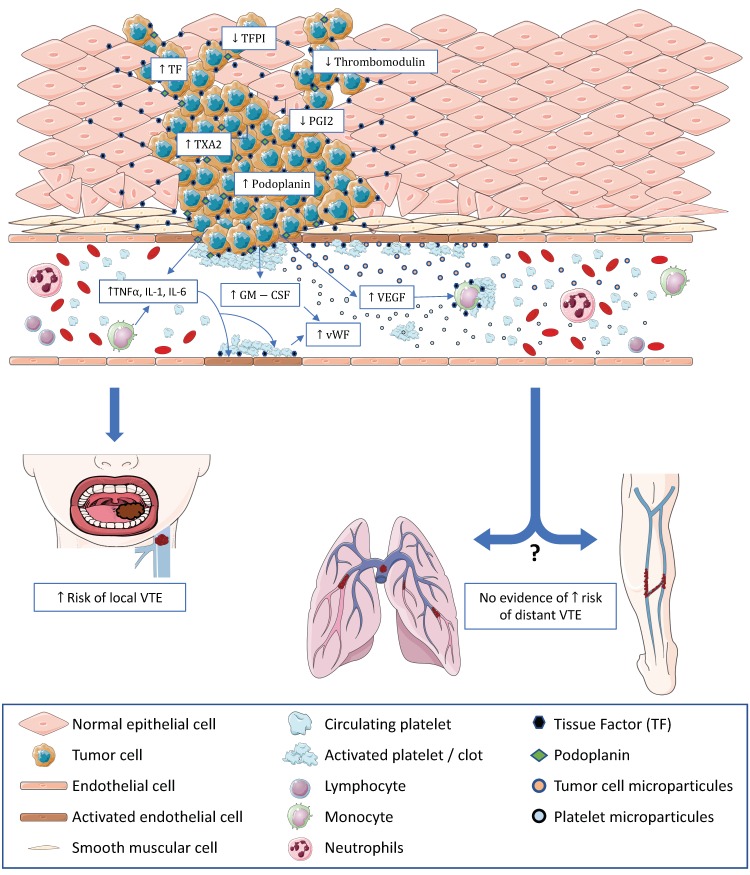
Potential mechanisms involved in thrombosis associated with H&N cancer and their consequences on the local and at-distance VTE risk. Some procoagulant molecules are overexpressed by tumor cells and in the tumor microenvironment including TF, podoplanin, and TXA2. In contrast, there is a downregulation of anticoagulant molecules, such as PGI2, TFPI and TM. Procoagulant MPs from tumor cells and from platelets are released. Procoagulant cytokines such as TNFα, IL1, and IL6 are secreted by immune cells or directly by tumor cells. Platelet activation is essentially supported by TF expression on tumor cells, activated endothelial cells, monocytes and potentially MPs. All these described mechanisms seem to be involved in elevating the risk of VTE in the areas adjacent to the tumor and might be in accordance with the clinical findings. However, it would be extremely difficult to draw conclusions about the influence of these mechanisms on the distant risk of VTE since our clinical review showed a very low incidence of pulmonary embolism and phlebitis associated with H&N cancer.

The physiopathogenesis of thrombosis associated with H&N cancer is complex and involves both clinical and biological factors. Our clinical review highlighted that H&N cancer may be associated with a very low rate of VTE, and most of time, H&N cancer was evaluated to have the lowest risk of all studied cancer localizations, and in some studies, the risk was even less than that of hospitalized patients without malignancies. However, we also reported that, notably, no strong factual evidence exists due to the presence of recurrent biases and the fact that some studies had contradictory results. Our review reported that biologically, H&N cancer is associated with most mechanisms found in cancers associated with a high thrombosis risk, including the following: modified thrombosis and fibrinolysis mechanisms, expression of procoagulant proteins, liberation of procoagulant MPs, and production of procoagulant cytokines. Taken together, these facts lead to a paradoxical conclusion: H&N cancer has the biological features of a cancer with a high risk of thrombosis but has a clinical thrombosis risk that is estimated to be very low. We propose the following hypotheses to explain this contradiction (Figure 1):

There are flaws in many of the clinical studies that have assessed the thrombosis risk associated with H&N cancer, and many studies might underestimate the risk.

H&N tumors show similar features to tumors with a high thrombosis risk, but H&N tumors only show local procoagulant abilities without any effects on distant VTE (phlebitis and pulmonary embolism).

Undiscovered mechanism(s) may exist, which could balance the potential thrombotic power of H&N tumors. We consider this last hypothesis to be the least unlikely.

The use of biological tests directly based on the molecular features of coagulation might be useful in validating these hypotheses and in determining the real thrombosis risk associated with H&N cancer. These tests would be an assessment of the thromboelastographic profile; the utility of which has been reported for some cancers, including those of the digestive tract and liver, and multiple myeloma [162,163,164]. These tests can also study thrombosis in vivo, especially in real time by using intravital microscopy procedures [161]. 

## Figures and Tables

**Table 1:** Incidence of H&N cancer diagnosis following VTE.

First Author	Year	Type of Study	Population	Number of Patients	Median Follow-up	Number and per Centage of H&N Cancer/All Diagnose Cancers	Rank H&N/Other Tumor Site	Ref.
Prandoni	1992	Prospective cohort study	Patient with DVT (deep vein thrombosis) (unprovoked or secondary to surgery or fracture)	250	2 years	0/13–**0%**	undefined	[23]
Piccioli	2004	Prospective clinical study	Patient with first idiopathic VTE (DVT or PE)	201	2 years	0/24–**0%**	undefined	[24]
Trujillo- Santos	2007	Case control study	Patient with VTE (DVT or PE) and occult cancer diagnose	14,623	3 month	1/178–**0.56%** Larynx	15/15	[25]
Sørensen	2012	Cohort study	Patient with superficial and deep VT and PE	77,247	15 years	35/6329–**0.55%** Larynx	25/25	[26]
Petterson	2015	Retrospective cohort study	Patient with VTE (DVT and PE) n = 1417	1417	13 years	5/345–**1.45%**	22/23	[27]
Robin	2016	Experimental prospective study	Patient screened by TEP/CT following DVT and PE	399	3 years	0/25–**0%**	undefined	[28]
Sun	2016	Retrospective case control study	Patient with unprovoked VTE	27,751	10 years	98/27751–**0.35%**	17/17	[29]
Sandén	2017	Retrospective cohort study	Patient with diagnostic of VTE (primary, secondary, unprovoked and following surgery or fracture)	7854	5 years	3/499–**0.6%**	12/13	[30]
Jara-Palomares	2017	Case control study	Patient with VTE	5863	2 years	5/444–**1.13%**	13/14	[31]
Delluc	2018	Prospective cohort study	Patient with DVT and PE	526	2 years	0/26–**0%**	undefined	[32]

**Table 2:** Incidence of VTE in patients with diagnosed with H&N cancer and a comparison to that of the other cancer localizations.

First Author	Year	Type of Study	Population	Number of Patient	Median Follow-up	Number and per Centage of VTE in H&N Cancer	Ranked Risk Compared to Other Cancer Localizations	Ref.
Levitan	1999	Retrospective cohort study	Patient with diagnosed cancer	1,211,944	6 years	35 VTE/20924–**0.16%**	18/18	[33]
Sallah	2002	Retrospective cohort study	Patients with solid tumor 1	1041	7 years	3 VTE/96–**3.125%**	11/11	[34]
Khorana	2006	Retrospective cohort study	Patient hospitalized with neutropenic cancer	66,106	7 years	44 VTE/1606–**2.74%**	21/21	[35]
Stein	2006	Retrospective cohort study	Patients hospitalized with cancer	40,787,000	20 years	<5000 VTE/849000–**<0.6%**	19/19	[36]
Khorana	2007	Retrospective cohort study	Patient with cancer	1,015,598	8 years	713 VTE/50898–**1.4%**	21/21	[37]
Paneesha	2010	Retrospective linkage cohort study	Patient hospitalized with cancer and/or VTE	39,618	3 years	Data not available, but elevated	2/18	[22]
Walker	2012	Cohort study	Patient with and without cancer	660,410	2 years	35 VTE/2078–**1.68%**	24/26	[38]
Chew	2015	Retrospective cohort study	Patient with diagnose cancer	43,855	7 years	40 VTE/4390–**0.91%**	21/23	[39]

**Table 3:** Incidence of VTE in patients with H&N cancer following treatment (surgery), an analysis of specific studies.

First Author	Year	Study Design	Population	Number of Patient	Median Follow-up	Number and per Centage of VTE in H&N Cancer	Ref.
Innis	2009	Retrospective review study	Patients following otolaryngological surgery with and without malignancy	6122	5 years	5 VTE/542–**0.92%**	[40]
Hennessey	2012	Retrospective cross sectional study	Patients following H&N cancer surgery	93,663	5 years	1860 VTE /93663–**2%**	[41]
Thai	2013	Retrospective review study	Patients following a > 4h00 H&N cancer surgery	134	2 years	2 (confirmed-8 (suspected) VTE/134–**1.4**–**5.8%**	[42]
Gavriel	2013	Retrospective cohort study	Patients following a H&N cancer surgery, with and without chemoprophylaxis	1018	5 years	0 VTE/1018–**0%** (both cohort)	[43]
Clayburgh	2013	Prospective cohort study	Patients following H&N cancer surgery	100	1 month	8 VTE /100–**8%**	[44]
Lodders	2015	Retrospective cohort study	Patients following oral cavity cancer surgery	233	5 years	1 VTE/233–**0.41%**	[45]
Ali	2015	Retrospective cohort study	Patients following H&N cancer surgery	413	8 years	12 VTE/413–**2.9%**	[46]
Kakei	2016	Retrospective descriptive study	Patients following oral cavity cancer surgery with simultaneous reconstruction	133	7 years	35 VTE/133–**26.3%**	[47]
Wang	2017	Retrospective descriptive study	Patients following oral cavity and maxillary cancer surgery	9724	4 years	14 VTE/9724–**0.14%**	[48]

## Abbreviations

VTE	venous thromboembolism
H&N	head and neck
SCC	squamous cell carcinoma
MPs	microparticules
TF	tissue factor
TFPI	tissue factor pathway inhibitor
OSCC	oral squamous cell carcinoma
ASCO	American Society of Clinical Oncology
LMWH	low molecular weight heparin
VKA	vitamin K antagonist
tPA	tissue plasminogen activators
uPA	urokinase plasminogen activators
PAI	plasminogen activator inhibitor
TAFI	thrombin activated fibrinolysis inhibitor
ELISA	enzyme-linked immunosorbent assays
TXA2	thromboxane A2
PGI2	prostacyclin
vWF	von Willebrand factor
TM	thrombomodulin
TNF	tumor necrosis factor
IL	interleukin
VEGF	vascular endothelial growth factor
GM-CSF	granulocyte-macrophage colony-stimulating factor
EGF	epidermal growth factor
PDGF	platelet-derived growth factor
TGFβ	transforming growth factor
